# Tellurium-Doped Bioactive Glass Induces Ferroptosis in Osteosarcoma Cells Regardless of FSP1

**DOI:** 10.3390/antiox13111327

**Published:** 2024-10-30

**Authors:** Elżbieta Pańczyszyn, Mari Lallukka, Mara Gagliardi, Valentina Saverio, Romina Monzani, Marta Miola, Enrica Verné, Marco Corazzari

**Affiliations:** 1 Center for Translational Research on Autoimmune and Allergic Disease (CAAD), Department of Health Science, University of Piemonte Orientale, 28100 Novara, Italy; elzbieta.panczyszyn@uniupo.it (E.P.); mara.gagliardi@uniupo.it (M.G.); valentina.saverio@uniupo.it (V.S.); romina.monzani@uniupo.it (R.M.); 2 Applied Science and Technology Department, Politecnico di Torino, 10129 Turin, Italy; mari.lallukka@polito.it (M.L.); marta.miola@polito.it (M.M.); erica.verne@polito.it (E.V.); 3 Interdisciplinary Research Center of Autoimmune Diseases (IRCAD), University of Piemonte Orientale, 28100 Novara, Italy

**Keywords:** ferroptosis, tellurium, osteosarcoma, FSP1, bioactive glass

## Abstract

Human osteosarcoma (OS) is a rare tumor predominantly affecting long bones and characterized by a poor prognosis. Currently, the first line of intervention consists of the surgical resection of primary tumors combined with radiotherapy and chemotherapy, with a profound impact on the patient’s life. Since the surgical removal of OS frequently results in a large resection of bones, the use of biomaterials to sustain the stability of the remaining tissue and to stimulate bone regeneration is challenging. Moreover, residual neoplastic cells might be responsible for tumor recurrence. Here, we explored the potential of tellurium-ion-doped bioactive glass as a novel therapeutic intervention to both eradicate residual malignant cells and promote bone regeneration. Bioactive glass (BAG) has been extensively studied and employed in the field of regenerative medicine due to its osseointegration properties and ability to improve bone tissue regeneration. We found that the incorporation of tellurium (Te) in BAG selectively kills OS cells through ferroptosis while preserving the viability of hBMSCs and stimulating their osteodifferentiation. However, the mechanism of Te toxicity is still unclear: (i) Te-BAG generates lipid-ROS through LOXs activity but not iron overload; (ii) Te-dependent ferroptosis is mediated by GPX4 down-regulation; and (iii) the anti-ferroptotic activity of FSP1 is abrogated, whose expression confers the resistance of OS to the canonical induction of ferroptosis. Overall, our data show that Te-doped bioglass could represent an interesting biomaterial with both pro-ferroptotic activity towards residual cancer cells and pro-osteoregenerative activity.

## 1. Introduction

The recently identified iron-dependent non-apoptotic form of cell death known as ferroptosis is being actively studied as a promising new frontier in anticancer therapy [1,2,3]. This interest is largely due to the increased sensitivity of cancer cells to ferroptosis, especially in light of their resistance to apoptosis—both intrinsic and acquired—which is the primary mode of cell death triggered by conventional cancer treatments [4]. Additionally, the ongoing debate regarding the interaction between ferroptosis and the immune system, which could significantly aid in tumor eradication, further enhances its potential therapeutic relevance in this area [5,6].

While the modulation of cellular iron metabolism [7]—resulting from increased iron uptake [8] and/or the enhanced mobilization of intracellular iron storage through ferritinophagy [9]—is regarded as a crucial step in boosting the intracellular labile iron pool (LIP) [10] responsible for lipid peroxide generation, other mechanisms are also at play. These include the activation of iron-dependent enzymes such as lipoxygenases (LOX) [1,2] and cytochrome P450 oxidoreductase (POR) [4,5].

Regardless of how they are generated, lipid peroxides (lipid-ROS) serve as the primary mediators of this form of cell death. This is largely due to their high reactivity and their incorporation into cell membranes as phospholipids (primarily PE-OOH) [11], which compromises membrane structure and function, ultimately leading to cell death. However, the precise molecular mechanisms underlying this process remain unclear [6].

On the other hand, cells evolved mechanisms to avoid the accidental and/or improper induction/execution of ferroptosis, mainly focused on lipid-ROS detoxification, with many cancer cells using those mechanisms to efficiently escape from ferroptosis execution. In this context, the signaling pathway linking the membrane glutamate/cystine antiporter system (system XC^-^) and the glutathione peroxidase 4 (GPX4) [12] represent the main anti-ferroptotic system, through which cystine intake is used to produce GSH that is used by GPX4 to reduce lipid peroxides [3,13]. The latter molecules can also be alternatively destroyed by (i) the GCH1-dependent BH4/BH2 cycle or (ii) the FSP1-dependent reduced CoQ1 [14]. Lipid-ROS can also be targeted by (iii) members of the aldo-keto reductase superfamily of enzymes (AKRs), thus blocking the execution phase of ferroptosis [2,15], while (iv) the ESCRT-III system efficiently repairs the lipid-peroxidation-mediated membrane damages, thus conferring resistance to ferroptotic cell death [16]. In addition to the above-mentioned and well-characterized anti-ferroptotic mechanisms, others are emerging with important implications for the ferroptotic-based therapeutic treatment of tumors, such as the one regulated by TG2, although the molecular details are still being studied [17,18].

Osteosarcoma (OS) is a common malignant bone tumor mainly affecting children, adolescents, and young adults. It arises from osteoid and immature bone, often in the long bones’ metaphysis [19]. Despite treatment involving surgery and chemotherapy, OS has a high mortality rate and poor prognosis, with drug resistance being a significant issue [20]. Immune checkpoint inhibitors (ICIs) have shown promise in other cancers, but have yet to prove effective in OS [21]. Research to enhance understanding and find new treatment approaches for OS is crucial due to limited progress in improving survival rates. Indeed, we have recently discovered that the sensitivity to ferroptotic cell death by osteosarcoma cells is subordinate to the levels of FSP1, while inhibiting the expression or activity of this factor efficiently re-sensitizes cells to ferroptosis [22].

Importantly, the surgical removal of primary osteosarcoma often involves significant bone resection, making it challenging to use biomaterials that can support the stability of the remaining tissue while promoting bone tissue regeneration. Recently, the potential of ion-doped bioactive glass as a novel therapeutic approach has emerged, capable of both eradicating malignant cells and simultaneously facilitating bone tissue regeneration [23]. Bioactive glass (BAG) has been extensively researched and utilized in regenerative medicine due to its osseointegration properties and ability to enhance bone tissue regeneration [24].

Since the initial report of its bone-binding properties nearly 40 years ago, 45S5 Bioglass^®^, specifically, has been widely studied for biomedical applications. A key feature of this material is its bioactivity, which allows it to release functional ions such as Ca^2+^ and PO_4_^3−^ upon contact with biological fluids. This process promotes the formation of a layer of nanocrystalline hydroxyapatite (HA) on the glass surface, demonstrating a strong affinity for living tissue. Additionally, these ionic dissolution products are well known to effectively enhance the differentiation of bone-forming cells and the mineralization of the extracellular matrix [25].

Furthermore, the incorporation of inorganic ions like silver (Ag^+^), copper (Cu^2+^), and zinc (Zn^2+^) into BAG structures provides specific biological functions, thereby improving their efficacy [24]. In this study, we demonstrate that the incorporation of tellurium (Te) into BAG results in the selective killing of osteosarcoma cells through ferroptosis while preserving the viability of human mesenchymal stem cells (hBMSCs) and promoting their osteodifferentiation. Although the molecular mechanisms underlying Te toxicity are not yet fully understood, our findings suggest that Te-BAG induces lipid peroxidation without requiring iron overload, potentially impacting the functionality of FSP1 directly.

## 2. Materials and Methods

### 2.1. Cell Culture and Treatments

Human osteosarcoma cell lines—U2OS, MG63, and HOS—were maintained in Dulbecco’s modified Eagle’s medium (DMEM; EuroClone, Milan, Italy) and supplemented with 10% fetal bovine serum (FBS; EuroClone, Milan, Italy), 2 mM L-glutamine (Merck, Milan, Italy), and 1% penicillin/streptomycin (Merck, Milan, Italy) at 37 °C in a humidified incubator with 5% CO_2_.

Human bone marrow-derived stem cells (hBMSCs) were kindly provided by Prof. P. Genever, University of York. hBMSCs were isolated from the bone marrow and then immortalized using hTERT lentiviral vectors (hBMSCs Y201). Cells were maintained in Dulbecco’s modified Eagle’s medium (DMEM; EuroClone, Milan, Italy) supplemented with 15% fetal bovine serum (FBS; EuroClone, Milan, Italy), 2 mM L-glutamine (Merck, Milan, Italy), and 1% penicillin/streptomycin (Merck, Milan, Italy) at 37 °C in a humidified incubator with 5% CO_2_. Cells were treated with RSL3 (Cayman Chemical, Ann Arbor, MI, USA) 0.5 µM, Ferrostatin-1 (Merck, Milan, Italy) 10 µM, AC-DEVD (Cayman Chemical, Ann Arbor, MI, USA) 10 µM, iFSP1 (BioVision, Abcam, Cambridge, UK) 6 µM, Deferoxamine (Merck, Milan, Italy) 10 µM, or Baicalein (Merck, Mialn, Italy) 20 µM, as indicated.

### 2.2. Bioactive Glass Synthesis

Silica-based bioactive glass doped with tellurium dioxide (TeO_2_) was developed as previously reported by Miola et al. [19] Two glass compositions (STe0 and STe5), as reported in Table 1, were developed by partially substituting SiO_2_ with TeO_2_. The amount of TeO_2_ was selected considering the potential toxic effect of this element, as previously described [26,27,28,29]. Briefly, the glasses were synthesized by melting the reactants in a Pt crucible at 1450 °C, pouring the melt in a brass mol and annealing them at 550 °C for 14 h. The obtained bars were cut in disc-shaped specimens (10 × 2 mm) and polished with abrasive SiC papers [19]. Biological characterization was performed on the discs. Specimens were heat-sterilized for 3 h at 180 °C and stored at room temperature prior to biological experiments.

### 2.3. Cytocompatibility Evaluation

The cytocompatibility of the investigated bioactive glass was tested in an indirect exposure culture, as previously described [30]. Briefly, specimens of STe0 and STe5 were soaked in 1.5 mL of culture medium for 72 h at 37 °C to stimulate iron release [30]. Subsequently, osteosarcoma cells and hBMSCs were exposed to an STe0/STe5-conditioned medium for 72 h.

### 2.4. Cell Viability

Cell viability was measured using the AlamarBlue™ reagent (Bio-Rad, Hercules, CA, USA) according to the manufacturer’s instructions, as previously described [31]. Briefly, 15 × 10^3^ cells/well were plated in 24-well plates, treated as indicated. The cell medium was discarded and an appropriate amount of AlamarBlue reagent was added. Cells were incubated for 4 h, and fluorescence was monitored (530–560 nm excitation and 590 nm emission wavelengths) using a TECAN automation platform.

Fluorescein diacetate (FDA)/7AAD staining was used to identify and measure the percentage of live/dead cells [17]. Briefly, cells were incubated (15–20 min) with PBS containing FDA (7 pg/mL; Thermo Fisher Scientific, Waltham, MA, USA) and 7AAD (50 ng/mL; Bio-Rad, Hercules, CA, USA) and 10.000 events were acquired using flow cytometry (FACSymphony, BD, Milan, Italy). The percentage of 7AAD positive and FDA negative cells was measured and is indicated as ‘cell death (%)’.

### 2.5. Real Time PCR (qPCR)

Total RNA was isolated using TripleXtractor reagent (Grisp, BioCell, Rome, Italy), and ExcelRT Reverse Transcriptase (Grisp, BioCell, Rome, Italy) was used to produce cDNA using 2 μg of total RNA. Quantitative PCR (qPCR) reactions were performed using the Excel-Taq FAST qPCR SybrGreen (Grisp, BioCell, Rome, Italy) and a CFX96 thermocycler (Bio-Rad, Hercules, CA, USA). Primer sequences were designed using the online IDT PrimerQuest Tool software (IDT; https://eu.idtdna.com/Primerquest/Home/Index (accessed on 2 February 2021)), and sequences are reported below (Table 2) [32].

The L34 mRNA level was used as an internal control and the comparative Ct method (ΔΔCt) was used for the relative quantification of gene expression.

### 2.6. Western Blotting Analysis

Proteins were isolated by using a RIPA Buffer supplemented with a protease inhibitor cocktail (Merck), and an equal amount of proteins (20 µg) were subjected to an SDS-PAGE and electroblotted onto nitrocellulose membranes (Bio-Rad, Hercules, CA, USA). Membranes were blocked for 1 h with 5% non-fat dry milk (Merck, Milan, Italy) in PBS plus 0.1% Tween20 (Merck, Milan, Italy) and incubated with the indicated primary antibodies in blocking solution overnight at 4 °C: anti-FSP1 (1:1000; ProteinTech, DBA, Milan, Italy), anti-NRF2 (1:1000; Cell Signaling Technology, Danvers, MA, USA), anti-FTH1 (1:1000; Santa Cruz Biotechnology, Dallas, TX, USA), anti-NCOA4 (ARA70; 1:1000, Santa Cruz Biotechnology, Dallas, TX, USA), anti-GPX4 (1:1000, Cell Signaling Technology, Danvers, MA, USA), anti-Tubulin (1:500; Santa Cruz Biotechnology, Dallas, TX, USA), and anti-GAPDH (1:500; Santa Cruz Biotechnology, Dallas, TX, USA). Detection was achieved using horseradish peroxidase (HRP)-conjugated secondary antibodies (1:5000; Jackson ImmunoResearch, West Grove, PA, USA) and visualized using SuperSignal West Pico Plus (Thermo Fisher Scientific, Waltham, MA, USA). Images were acquired using a ChemiDoc Touch Imaging System (Bio-Rad, Hercules, CA, USA) and analyzed using Image Lab 5.0 software (Bio-Rad, Hercules, CA, USA) [33]. Quantification (densitometric analysis) was performed by using Image Lab 5.0 software.

### 2.7. Detection of Intracellular Fe^2+^

A BioTracker FerrOrange Live Cell Dye (Merck, Milan, Italy) was used to detect intracellular labile ferrous (Fe^2+^) ions according to the manufacturer’s protocol, as previously described [22]. Briefly, 15 × 10^3^ cells/well U2OS were plated in 24-well plates and then exposed to STe5 or STe0 for 48 h. The cells were then washed twice with PBS and treated with 1 uM FerrOrange in DMEM without FBS for 30 min at 37 °C. After incubation, FerrOrange was removed by washing cells twice with PBS, and fluorescent signal was recorded using a THUNDER 3D Cell Imager (Leica, Wetzlar, Germany). Quantification was performed by using ImageJ 1.54k software.

### 2.8. Immunofluorescence

Samples were washed with cold PBS and fixed in PFA (Merck, Milan, Italy) 4% for 15 min at 4 °C, permeabilized with 0.5% Triton X-100 (Merck, Milan, Italy) in cold PBS for 10 min at 4 °C, washed twice, and incubated with 10% donkey serum (Jackson ImmunoResearch, West Grove, PA, USA) plus 0.05% Triton X-100 in cold PBS for 30 min at 4 °C. Next, the slides were washed and incubated with anti-FSP1 (1:300, ProteinTech, DBA, Milan, Italy) primary antibody in 1% donkey serum plus 0.05% Triton X-100 in cold PBS for 1 h at 4 °C. After washing, the slides were incubated with appropriate secondary antibodies (Jackson ImmunoResearch, West Grove, PA, USA) 1:500 in 1% donkey serum plus 0.05% Triton X-100 in cold PBS and incubated for 1 h at 4 °C.

Coverslips were mounted onto glass using ProLong Gold Antifade with DAPI mounting solution (Thermo Fisher Scientific, Waltham, MA, USA). Images were acquired using an SP8 confocal microscope (Leica) [32].

### 2.9. Statistical Analysis

The experiments were performed in triplicate and repeated at least three times. Statistical analyses were performed using the GraphPad software (GraphPad Software; GraphPad Prism 6). Student’s *t*-test or ANOVA was used to determine the statistical significance. A *p*-value of equal to or less than 0.05 was considered significant. mRNA expression levels are represented as ‘fold change’ relative levels. Histograms represent mean ± SD; **** *p* < 0.0001; *** *p* < 0.001; ** *p* < 0.01; * *p* < 0.05; ns = non-significant.

## 3. Results

### 3.1. Selective Toxicity of Tellurium-Doped Bioactive Glass to Osteosarcoma Cells and Osteoinductive Effect on hBMSCs

**Figure 1:**
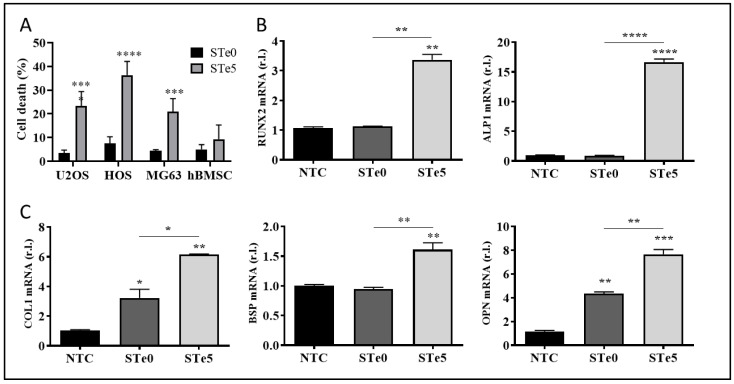
STe5 kills OS cells while inducing osteogenic markers in hBMSCs. (**A**) The indicated OS cell lines and hBMSCs were exposed to balk BAGs-(STe0) or tellurium-doped BAGs-(STe5)-conditioned media, and cell death was evaluated after 72 h. (**B**,**C**) hBMSC were exposed to STe0- or STe5-conditioned media and the expression of the indicated pro-osteogenic markers was evaluated by qPCR after 72 h. Experiments were performed in triplicate and repeated three times. Histograms represent the mean ± SD; * *p* < 0.05; ** *p* < 0.01; *** *p* < 0.001; **** *p* < 0.0001.

We investigated the osteoinductive and anticancer properties of silica-based bioactive glass containing a low amount of tellurium dioxide (TeO_2_), as this active element and its compounds are currently being discovered as novel and valid cancer therapeutics [34]. To this end, and to confirm our previous results on cytocompatibility [19], we treated our panel of human osteosarcoma cells (U2OS, MG63, and HOS) and human bone marrow stem cells (hBMSC) with a medium conditioned with the dissolution products of tellurium-doped BAG (STe5) or base BAG composition (STe0), and cell viability was measured after 72 h. Data reported in Figure 1A indicate that the addition of tellurium to BAG efficiently killed osteosarcoma cells, increasing the number of dead cells to 25% for U2OS, 36% for HOS, and 24% for MG63, while no significant deleterious effect was observed on hBMSCs. Thus, these results suggest the potential selective toxicity of Te-doped bioactive glass (Te-BAG) to bone cancer cells.

Next, we evaluated the levels of osteogenic differentiation markers, such as RUNX2, ALP, COL1, OPN, and BSP1, in hBMSC exposed to media conditioned with ionic dissolution products of STe0 and STe5 by qPCR. Interestingly, the enhanced mRNA expression of the indicated osteogenesis markers was evident after 72 h of culture with extracts derived from tellurium-doped bioactive glass (STe5). In contrast, samples without tellurium (STe0) exhibited lower osteostimulation effects on hBMSC (Figure 1B,C). Of note, RUNX2 and ALP are consistently referred to as early transcription factors and are predominantly expressed in preosteoblasts and osteoblasts [35]. Collectively, our results indicated that the expression of genes specific to osteoblasts was significantly enhanced by the medium conditioned by STe5, thus suggesting the potential use of Te-BAG as a platform for bone regeneration and targeted therapy for osteosarcoma.

### 3.2. Tellurium-Doped Bioactive Glass Induces Ferroptosis-Mediated OS Cell Death

**Figure 2:**
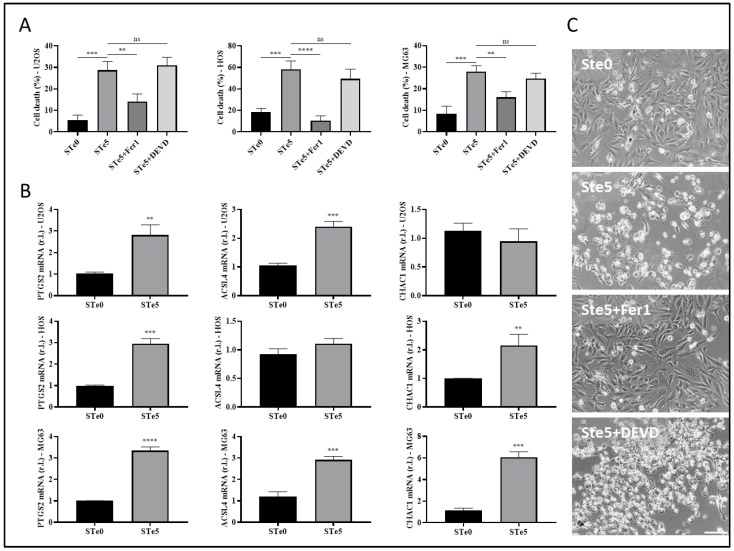
Tellurium-doped BAG specifically induces ferroptosis in OS cells. (**A**) The indicated OS cell lines (U2OS, MG63, and HOS) were exposed to a medium conditioned by STe5 alone or in the presence of the ferroptosis inhibitor Fer1 (10 µM) or the apoptosis inhibitor AC-DEVD (10 µM), and cell viability was evaluated after 48 h. The STe0-conditioned medium was used as a control. (**B**) U2OS (upper panels), HOS (middle panels), or MG63 (bottom panels) were exposed to STe0- or STe5-conditioned media and the expression of the pro-ferroptotic markers PTGS2, ACSL4, or CHAC1 was evaluated by qPCR after 72 h. (**C**) HOS were exposed to a medium conditioned by STe0 or STe5 alone or in the presence of Fer1 (10 µM) or AC-DEVD (10 µM), and cell morphology was evaluated by light microscopy (phase contrast) at 72 h. The appearance of cell membrane blebbing, characteristic of cells dying through ferroptosis, is evidenced by green arrows (scale bar 100 µm). Experiments were performed in triplicate and repeated three times. Histograms represent the mean ± SD; ** *p* < 0.01; *** *p* < 0.001; **** *p* < 0.0001; ns: not statistically significant.

Apoptosis has long been considered a deliberate mechanism of regulated cell death (RCD), and the pathways involved in this process have been extensively studied in various types of tumor cells. The induction of apoptosis is recognized as a prominent therapeutic approach for eliminating cancer cells [36,37]. However, accumulating evidence has proven that anti-tumor strategies based on the induction of the non-apoptotic form of RCD known as ferroptosis are a promising direction for addressing certain challenges in cancer therapy [2,15,36]. Therefore, to identify the process underlying the observed toxicity of tellurium-doped bioactive glass to OS cells, we evaluated the involvement of both apoptosis and ferroptosis. To this end, the three OS cell lines were exposed to STe5 in the presence or absence of the ferroptosis inhibitor ferrostatin-1 (Fer1) or the apoptosis inhibitor AC-DEVD [38] for 72 h, while STe0 was used as a control. Data reported in Figure 2A clearly show that the cytotoxic effect of STe5 was consistently inhibited by ferrostatin-1 (STe5 + Fer1), while AC-DEVD was ineffective.

Subsequently, the evaluation of the ferroptotic markers PTGS2, ACSL4, and CHAC1, performed by qPCR, supported the induction of this form of cell death in cells exposed (48 h) to a medium conditioned with STe5 compared to samples without tellurium (STe0; Figure 2B). Additionally, ferroptotic cell death induced by STe5 was further confirmed in HOS cells by the typical morphological features characterized by plasma membrane bubbling (Figure 2C) [39]. Importantly, Te-dependent morphological change was completely prevented by the concomitant exposure to Fer1, while AC-DEVD was ineffective (Figure 2C).

Hence, our results confirmed the anticancer properties of tellurium-doped bioactive glass, indicating that ferroptosis is the main pathway of RCD responsible for its cytotoxic effect on osteosarcoma cells.

Of note, Te-BAG also efficiently induced ferroptosis in U2OS cells, a cell line that we recently identified as the most resistant to the conventional induction and execution of ferroptotic cell death, compared to both MG63 and HOS, due to the enhanced expression of FSP1 [22]. Thus, U2OS cells were used for further investigations aimed at identifying potential molecular targets and/or cellular mechanisms underlying the cancer-cell-specific toxic effect of Te-BAG, which is capable of re-sensitizing OS cells through ferroptosis.

### 3.3. Role of Iron in Te-BAG-Induced Ferroptosis

**Figure 3:**
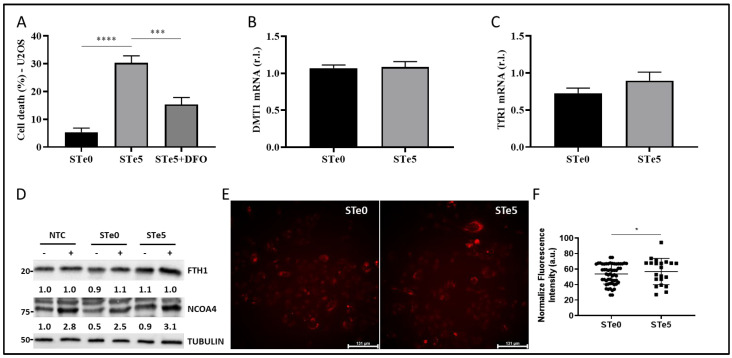
Te-BAGs-induced ferroptosis does not affect iron metabolism. (**A**) U2OS cells were exposed to a medium conditioned by STe5 alone or in combination with iron chelator deferoxamine (DFO, 10 µM), and cell viability was evaluated at 72 h. The STe0-conditioned medium was used as a control. The expression of divalent metal transporter 1 (DMT1; (**B**) or transferrin receptor 1 (TfR1; (**C**) was evaluated in cells treated as in A at 48 h by qPCR. (**D**) The expression of the ferritinophagy markers ferritin heavy chain 1 (FTH1) and nuclear receptor coactivator 4 (NCOA4) was evaluated in U2OS cells exposed to an STe5- or STe0-conditioned medium, alone or in combination with autophagy inhibitor bafilomycin A1 (BAF), by Western blotting. Tubulin was used as a loading control, and the results of the densitometric analysis were reported for each corresponding band. (**E**) U2OS cells were exposed to tellurium-doped bioactive glass-(STe5) or a basal composition-(STe0)-conditioned medium for 48 h, and the intracellular Fe^2+^ was evaluated by confocal microscopy (in red) using FerrOrange probe. Scale bar = 131 µm. FerroOrange fluorescence quantification was performed by using ImageJ 1.54k (**F**). Experiments were performed in triplicate and repeated three times. Histograms represent the mean ± SD; * *p* < 0.05; *** *p* < 0.001; **** *p* < 0.0001.

Recently, Liu and co-workers reported that macrophages exposed to tellurium compounds exhibited ferroptosis, the induction of which was associated with the degradation of ferritin heavy chain 1 (FTH1), the primary protein responsible for intracellular iron storage, through an autophagy-based process known as ferritinophagy [40,41]. The occurrence of intracellular iron overload, a hallmark of ferroptosis, resulting from this process subsequently promotes the production of lipid peroxides [42]. To investigate whether Te-BAG triggers ferroptosis in OS cells by disrupting intracellular iron homeostasis, U2OS cells were exposed to a medium conditioned with STe5 in the presence of the iron chelator and ferroptosis inhibitor deferoxamine (DFO) [43]. Remarkably, DFO significantly decreased the cytotoxic effect of STe5, as shown in Figure 3A, indicating a potential involvement of iron in STe5-stimulated lipid peroxidation.

To gain a deeper insight into the involvement of iron-dependent pathways in Te-BAG-induced ferroptosis, we also evaluated the expression profiles of key signaling molecules involved in iron uptake and transport, such as TfR1 and DMT1 [22,44]. However, any notable alteration in the expression of these signaling molecules was observed upon the exposure of U2OS cells to the STe5-conditioned medium when compared to samples derived from basal BAG composition (STe0; Figure 3B,C, evaluated by qPCR). Next, we explored the potential of Te-BAG to induce ferroptosis through the activation of ferritinophagy, a type of autophagy relying on the NCOA4-mediated and lysosome-dependent degradation of ferritin-iron aggregates. Regardless, our findings showed no significant alterations in NCOA4 or FTH1 protein levels that would suggest the activation of this process, in cells exposed to STe5 compared to STe0, when autophagy was inhibited by bafilomycin A1 (BAF; Figure 3D) [45]. Therefore, our data indicate that, although iron seems to be involved in the execution of the ferroptosis of OS cells exposed to tellurium, no evident alteration of Fe metabolism was observed. This conclusion is further sustained by measuring the intracellular iron concentration by staining cells with FerrOrange. Indeed, although intracellular iron accumulation is frequently observed in cells dying through ferroptosis, our analysis revealed no increase in free iron content in U2OS cells exposed to STe5 (Figure 3E). Collectively, our findings suggest that the mechanism underlying Te-BAG-induced ferroptosis does not involve the disruption of cellular iron uptake and storage or intracellular iron overload.

### 3.4. Te-BAG-Induced Ferroptosis Circumvents the Inhibitory Activity of FSP1

**Figure 4:**
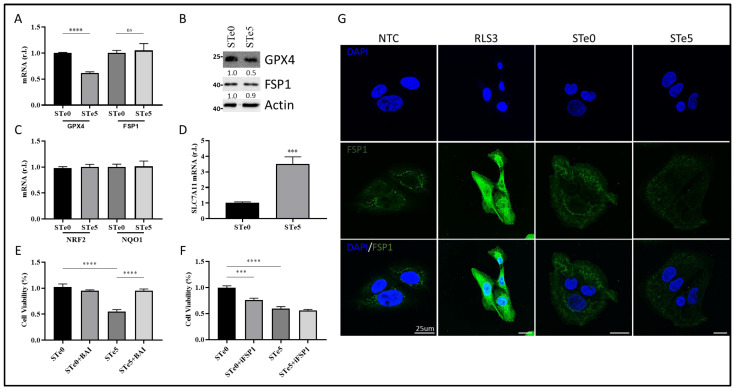
Te-BAG-induced ferroptosis regardless of FSP1, but ignited by GPX4 down-regulation. U2OS were exposed to the STe5- or STe0-conditioned medium (48 h) and the levels of GPX4 and FSP1 mRNA (**A**) or protein (**B**) were evaluated (and quantitated) together with the mRNA levels of NRF2, NQO1 (**C**), or SLC7A11 (**D**) by qPCR or Western blotting analysis (Actin was used as loading control). Cells were exposed to a medium conditioned by STe0 or STe5 alone or in the presence of Baicalein (BAI, 10 µM; (**E**) or FSP1 inhibitor (iFSP1, 10 µM; (**F**), and cell viability was evaluated at 72 h. Finally, U2OS were exposed or unexposed to RLS3 (0.5 µM) or to the STe0- or STe5-conditioned medium, and FSP1 expression was evaluated by immunofluorescence (**G**). FSP1 was evidenced by anti-FSP1 antibody (green), while nuclei were stained with DAPI (blue). Scale bar 25 µm. Experiments were performed in triplicate and repeated three times. Histograms represent the mean ± SD; *** *p* < 0.001; **** *p* < 0.0001; ns = not significant.

Although the molecular basis of Te toxicity has not yet been fully determined, one of the potential mechanisms is the substitution of the sulfur group in various amino acids, leading to the formation of dysfunctional proteins. Moreover, based on the homologies observed in the characteristics of elements belonging to the same group as tellurium, such as selenium, another hypothesis on the potential mechanism of cytotoxicity depends on its ability to oxidize glutathione (GSH), which causes the accumulation of ROS [46] and, possibly, lipid peroxides. Indeed, Wu and colleagues recently reported the introduction of Te nanowires as an inorganic prodrug with the capability to selectively deplete glutathione and elevate ROS levels to lethal thresholds in cancer cells without inducing oxidative stress in normal cells [47]. Moreover, the disruption of redox homeostasis observed in the presence of Te compounds was also correlated with a decrease in NRF2 level [40]. Thus, we examined the expression of two key antioxidant factors, GPX4 and NRF2. Of note, ROS production/accumulation and the dysregulation of NRF2 and GPX4 expression/activity are also biomarkers of ferroptosis, with GPX4 directly involved in lipid-ROS demolition. Indeed, we observed decreased GPX4 expression in U2OS cells exposed to the STe5-conditioned medium, while no significant changes in NRF2 and direct target NQO1 expression were noted, in the same experimental conditions, compared to cells exposed to the STe0-conditioned medium (Figure 4A–C).

We also found increased expression of SLC7A11 in cells exposed to tellurium, as the cells try to counteract the reduced GPX4 activity by increasing GSH production (Figure 4D).

Importantly, the cytotoxic effect of STe5 was effectively abolished by concomitant exposure of cells (U2OS) to STe5 and baicalein (BAI; Figure 4E), which inhibits lipoxygenase (LOX) activity [15,48,49]. These data confirm the key role played by lipid-ROS in Te-BAG-induced ferroptosis.

Next, we examined the role of FSP1 in the ferroptotic process elicited by tellurium-doped bioglass. Specifically, we analyzed the reason why this critical antioxidant pathway, which contributes to the cellular resistance of U2OS cells under conventional ferroptosis induction [22], is insufficient to protect against the process triggered by Te. In fact, as reported in Figure 4F, the simultaneous administration of STe5 and iFSP1, which specifically inhibits FSP1 activity [22,50,51], did not result in a significant increase in cell sensitivity to ferroptosis execution (Figure 4F). Notably, the effect of iFSP1, which sensitizes cells to ferroptotic cell death, was observed in cells exposed to basic materials (STe0), implying that tellurium may interfere with FSP1 activity. Finally, while FSP1 expression was enhanced in U2OS cells exposed to RLS3, as expected [22], no significant changes were observed in cells exposed to the STe5-conditioned medium compared to the STe0-conditioned medium (Figure 4G).

## 4. Discussion

Osteosarcoma is a rare disease that impacts bone tissue, with primary treatment involving extensive surgical resection along with chemotherapy, radiotherapy, and immunotherapy. Commonly used drugs include methotrexate, cisplatin, doxorubicin, and ifosfamide [52,53]. Despite advancements, the 5-year survival rate hovers around 70%, facing challenges such as side effects from high-dose chemotherapy and drug resistance [54]. Targeted therapies that focus on specific molecular and cellular pathways are emerging as a promising option, while immune checkpoint inhibitors like mifamurtide show potential for enhancing survival rates [20,55,56,57,58].

However, fully eliminating cancer cells is extremely challenging, even when substantial amounts of the surrounding tissue are removed during surgery. It is important to note that surgical resection often leads to significant long-term challenges for patients, who are often quite young. Additionally, tumor recurrence is a significant concern, likely due to residual cancer cells that remain undetectable [59,60]. Consequently, there is an urgent need for new therapeutic strategies to effectively eliminate the tumor while minimizing the impact on the affected bones.

In this context, developing new biomaterial formulations that combine osteogenic properties to promote tissue regeneration with anticancer activity to eliminate residual cancer cells could offer a powerful innovative treatment for these patients. Bioactive glass doped with inorganic ions presents promising therapeutic opportunities, as these elements not only enhance the known osteogenic properties of bioglass, but also introduce new biological functions [23]. For instance, tellurium-doped bioactive glass (Te-BAG) has been shown to be well tolerated by human bone marrow stem cells (hBMSCs) and consistently improves the pro-osteogenic properties of bioglass in vitro. As a result, this biomaterial could accelerate bone tissue regeneration and enhance the healing process. On the other hand, we found that the presence of Te confers pro-ferroptotic activity to doped BAG. Indeed, tellurium efficiently induced ferroptosis in osteosarcoma cell lines regardless of the levels of the anti-ferroptotic factor FSP1 we recently described as representing a valid biomarker of resistance [22]. Indeed, Te activity seems to rely on two levels: (i) to prevent FSP1 upregulation, which is observed in cells exposed to canonical pro-ferroptotic drugs (e.g., RLS3), and (ii) to interfere with its anti-ferroptotic activity.

Although further studies are required to fully elucidate the molecular mechanism through which Te abrogates the anti-ferroptotic properties of FSP1, its pro-ferroptotic activity seems to rely on the down-regulation of the main anti-ferroptotic factor GPX4 while increasing the expression of the upstream factor SLC7A11. The latter phenomenon is not surprising, since cancer cells respond to the reduced expression/activity of GPX4, increasing the uptake of cystine to enhance the production of GSH to be used by GPX4 to reduce lipid-ROS, thus counteracting ferroptosis execution. Moreover, although Te does not dysregulate iron metabolism, which typically increases LIP, a hallmark of ferroptosis and responsible for lipid peroxides generation, iron trapping abrogates Te-stimulated ferroptosis. The latter, together with the inhibition of ferroptosis stimulated by tellurium through concomitant exposure to baicalein, strongly indicates the involvement of LOXs. Indeed, these are iron-dependent enzymes actively producing PUFA-OOH, thus participating in ferroptosis, whose activity can be inhibited by baicalein and/or chelating intracellular iron [2,15].

The present study is, of course, limited by using an in vitro approach, which lacks the complexity of whole tissues/organs. Therefore, the results need to be confirmed using in vivo models. Moreover, it would also be interesting to evaluate whether the ability of tellurium to compromise the anti-ferroptotic activity of FSP1 is strictly related to osteosarcoma or is a general feature of this transition element, which would have important implications in cancer therapy.

In conclusion, our work clearly shows that Te-BAGs might represent a new valuable opportunity in the clinical management of osteosarcoma due to their combined pro-osteogenic and pro-ferroptotic activities.

## Figures and Tables

**Table 1:** Nominal compositions of the investigated glasses.

	% mol	
	STe0	STe5
SiO_2_	48.6	43.6
Na_2_O	16.7	16.7
CaO	34.2	34.2
P_2_O_5_	0.5	0.5
TeO_2_	0.0	5.0

**Table 2:** Primer sequences.

Name/Gene ID	Sequence
*alp* [250]	GAGTATGAGAGTGACGAGAAAG/GAAGTGGGAGTGCTTGTATC
*bsp* [3381]	CAGAAGAGGAGGAGGAAGAA/CCCAGTGTTGTAGCAGAAAG
*col1* [1277]	GGATTCCAGTTCGAGTATGG/CAGTGGTAGGTGATGTTCTG
*dmt1* [4891]	GCTGTCTTCCAAGATGTAGAG/GGATGGGTATGAGAGCAAAG
*fsp1* [84883]	CCTGCCCTTCTCTCATCTTA/GTCCTCATAGGCCTGGATAG
*gpx4* [2879]	AGCTCTTCTGGGAAGTAGAC/CCTCCCTGTACCACATCTAT
*l34* [6164]	GTCCCCGAACCCTGGTAATAGA/GGCCCTGCTGACATGTTTCTT
*nqo1* [1728]	GGATGAGACACCACTGTATTT/CTCCTCATCCTGTACCTCTT
*nrf2* [4780]	CCTGCCCTTCTCTCATCTTA/GTCCTCATAGGCCTGGATAG
*opn* [6696]	CCCATCTCAGAAGCAGAATC/TGGCTTTCGTTGGACTTAC
*ptgs2* [5743]	GCCTGGTCTGATGATGTATG/GTATTAGCCTGCTTGTCTGG
*runx2* [860]	GAATGCCTCTGCTGTTATGA/GAAGACGGTTATGGTCAAGG
*slc7a11* [23657]	CTGGGTTTCTTGTCCCATATAA/GTTGCCCTTTCCCTCTATTC
*tfr1* [7037]	GTGAGGGATCTGAACCAATAC/TGGAAGTAGCACGGAAGA

## Data Availability

Data generated are reported in the article.
